# Exploring New Treatment Strategies for Endometriosis—Narrative Review

**DOI:** 10.3390/ijms27156750

**Published:** 2026-07-28

**Authors:** Katarzyna Błaszczak-Świątkiewicz, Michael Oettel

**Affiliations:** 1 Department of Pharmacology and Toxicology, Social Academy of Sciences, Sienkiewicza 9, 90-113 Łódź, Poland; 2 Independent Researcher, 90-113 Łódź, Poland

**Keywords:** endometriosis, selective progesterone modulators, dienogest, Asoprisnil, Vilaprisan, progesterone receptor, mesoprogestins, PAEC, EC313, progesterone resistance, endometriosis-associated pain

## Abstract

The pharmacological treatment of endometriosis using SPRMs requires improvement. The pure PR-agonist dienogest (DNG), a clinically established progestin, can serve as a reference compound for comparison. Its efficacy and safety have been well characterised in preclinical in vitro and in vivo studies, as well as in qualified pivotal clinical studies for the treatment of endometriosis. The pharmacodynamic profile of Dienogest (DNG) is compared with the experience of using the pure PR antagonist Vilaprisan and the mixed PR agonist/antagonist Asoprisnil to treat endometriosis. Unfortunately, the clinical development of Vilaprisan was suspended due to long-term toxicology findings in animals that require further clarification. The clinical development of Asoprisnil, however, was discontinued due to concerns regarding SPRM-associated endometrial changes, although the interpretation of PAEC has been debated. Additionally, another SPRM, Ulipristal acetate, has been associated with rare but serious cases of liver injury. However, Vilaprisan is primarily supported by fibroid-related clinical development, whereas Asoprisnil has limited Phase II endometriosis evidence. Nevertheless, this new mesoprogestin, Asoprisnil, has shown signs of tissue-selective activity in limited clinical and preclinical studies. Continuing the strategy of using mixed PR agonists/antagonists could inspire further drug development. The preclinical profile of the new mesoprogestin EC313 has been presented, showing a higher PR agonistic/antagonistic quotient than Asoprisnil, with comparatively elevated anti-endometriotic activity. Furthermore, EC313 increases the progesterone receptor isoform B/A ratio, but its clinical efficacy remains unproven as it is still in the early stages of development. Based on the preclinical data currently available, the next generation of mesoprogestins warrants further investigation for the treatment of endometriosis and related gynaecological disorders. This prompts consideration of whether there is potential to enhance the effectiveness of drug discovery strategies within this particular pharmacological class.

## 1. Introduction

Endometriosis (EM) and uterine leiomyomas (uterine fibroids; UF) are now among the most common benign gynaecological disorders in women of reproductive age. Fuldeore et al. [1] reported that approximately 65% of endometriosis patients in the United States (around 10% of women of childbearing age) underwent an endometriosis-related surgical procedure within one year of their initial diagnosis, with average costs ranging from $4.289 ± 3.313 for a diagnostic laparoscopy to $11.397 ± 8.747 for an abdominal hysterectomy. Rogers et al. [2] estimated the cost of EM treatment in the United States in 2009 to be $69.4 billion. Cardozo et al. [3] estimated the financial burden associated with uterine fibroids to be between $5.89 and $34.37 billion per year in the United States. Given this significant economic burden, pharmacological approaches are becoming increasingly meaningful, particularly for non-severe and mild cases. Optimising the medical therapy for these two disorders is one of the most challenging tasks for 21st-century drug research.

Since the etiopathogenesis of EM and UF is unclear in parts and is probably non-monocausal, discussions about possible causes are often controversial and cover many aspects. EM occurs in several different phenotypes, such as superficial or deeply invasive disease, and one mechanism may not account for all disease varieties. It is known that EM is mainly driven by excess oestrogen, including reduced inactivation of 17β-estradiol, whereas progesterone (P) plays an opposing role [4,5].

Progesterone resistance is believed to play a crucial role in the pathogenesis of EM. Endometriotic lesions demonstrate decreased expression of the PRA isoform and an absence of the PRB isoform compared to ectopic endometrium [6,7,8], whereas progesterone signalling is mainly assumed to be responsible for UF [9].

The search for useful druggable pharmacological targets for new drug discovery and development is complicated by different genetic and epigenetic pathways [4,10]. Konrad et al. [11,12] provided a valuable review of the etiopathogenesis of EM, including the lesser role of epithelial–mesenchymal transition.

Based on clinical trials conducted thus far in this particular area of women’s health, only a few new drugs have been authorised for sale. One of these is the pure progestin Dienogest (DNG), which has produced remarkable clinical results. DNG is approved for the treatment of endometriosis [13,14]. Other registered progestins are used off-label. In many regions, DNG has become a primary treatment for suppressing endometriosis. Additionally, DNG is effective against uterine fibroids [15,16] and adenomyosis [17,18,19,20]. The first antiprogestin, Mifepristone (RU 486), has been shown to be effective in treating endometriosis (EM) and adenomyosis [21]. However, due to its strong antiglucocorticoid effect, its use is limited, particularly in gynaecological applications.

In addition to the classical progestins and SPRMs such as Ulipristal acetate and Telaprostal (see concerns relating to its hepatotoxicity), the newer compounds Vilaprisan and mesoprogestin Asoprisnil have also demonstrated clinical therapeutic effectiveness in treating two very different diseases like endometriosis and fibroids, despite their significant pharmacodynamic differences.

As Ulipristal acetate has been associated with rare but serious cases of liver injury, some of which have required liver transplantation, the European Medicines Agency (EMA) has decided to restrict its use [22]. Following this development of SPRMs, another SPRM named Vilaprisan was discovered, with no serious toxicological symptoms observed during the clinical phase II study. This allowed it to progress to Phase III for endometriosis treatment. Unfortunately, all further clinical studies of Vilaprisan were suspended due to the appearance of drug toxicity signals in the long-term animal tests conducted in parallel with Phase III [13]. Next, a new subgroup of SPRMs, mesoprogestins, with PR-antagonistic and PR-agonistic activity, has been recognised [23]. Asoprisnil, a member of this subgroup, has attracted scientific attention due to its effectiveness in treating endometriosis and fibroids [24,25,26]. Unfortunately, typical human benign endometrial changes under SPRM therapy (progesterone receptor modulator changes, or PAEC) have been misinterpreted, resulting in the termination of further development of Asoprisnil [27]. Recently, EC313 has joined the mesoprogestin subgroup as a candidate for novel approaches to treating endometriosis, fibroids (including adenomyosis), heavy uterine bleeding and other women’s health disorders. Preclinical studies show that EC313 has a unique ratio of PR-agonistic to PR-antagonistic activities. It is highly effective in humanised in vivo models for both EM and UF [28]. Alongside progestins and antiprogestins, as well as mesoprogestins, several oral GnRH antagonists, including Elagolix, have entered clinical practice or late-stage development [13,29].

In order to optimise the steroid therapy for endometriosis and uterine fibroids, it is strategically helpful to compare the preclinical and clinical pharmacodynamics of the successful pure progestin DNG with those of the antiprogestin Vilaprisan and the mesoprogestin Asoprisnil. This comparison could lead to improvements in drug development strategies. Mifepristone and Ulipristal acetate are well known and their pharmacodynamic properties and clinical characteristics can be found in many places in the literature [5,13]. However, these two steroids were used as reference compounds in some of the studies cited below.

## 2. Chemistry

### 2.1. Dienogest (DNG)

Pure progestins are characterised by a diene structure between steroid rings A and B and a cyanomethyl group in the 17α-position of ring D. It has been suggested that the ethinyl group in the 17α-position of other 19-norprogestins may impact the interaction with the cytochrome P450 3A. For example, it may have a negative effect on haemostatic balance [30]. Consequently, DNG without a 17α-ethinyl group can reduce the risk of such an interaction. For instance, it can lower the likelihood of thrombosis [31,32].

### 2.2. SPRMs

**Figure 1:**
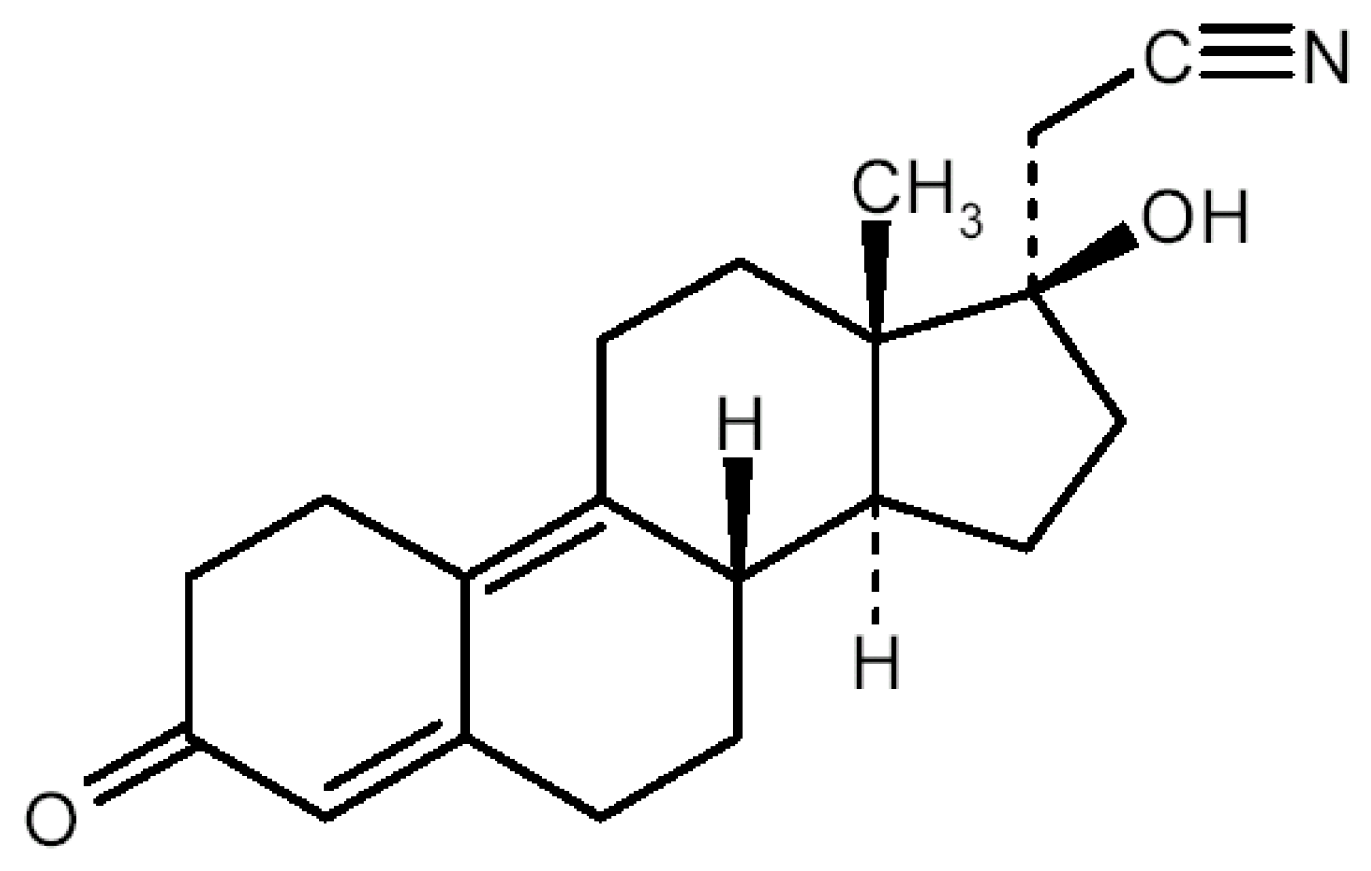
Structure of Dienogest (Lab Code STS 557), 17α-cyanomethyl-17β-hydroxy-estra-4,9 (10)-3-one. Molecular formula C_20_H_25_NO_2_. Molecular weight 311.42.

**Figure 2:**
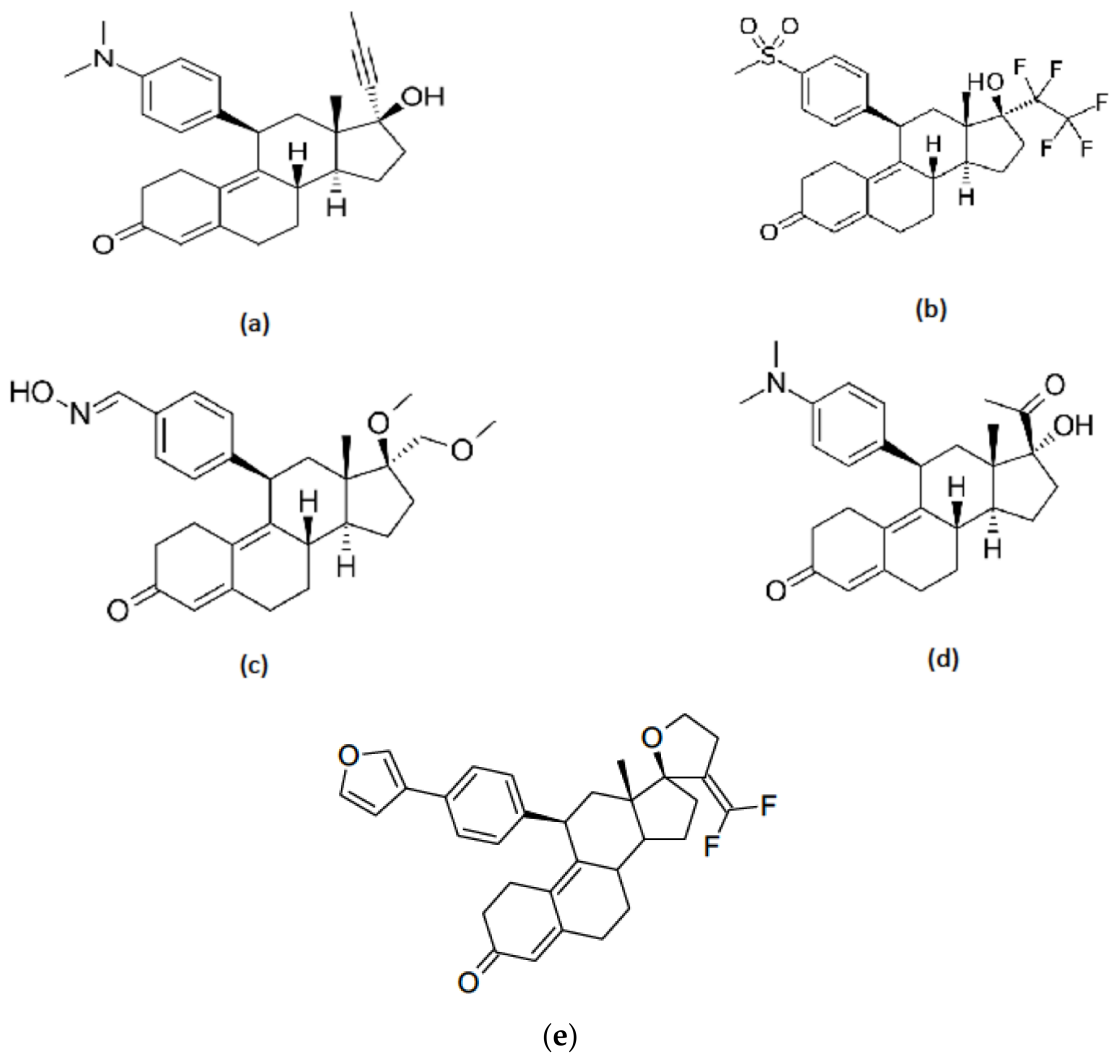
Structure of selected SPRMs: (**a**) Mifepristone (RU486), (**b**) Vilaprisan (BAY 1002670), (**c**) Asoprisnil (J-867), (**d**) Ulipristal acetate (CDB-2914) and (**e**) EC313.

All of the other PR agonists/antagonists presented here also possess a diene configuration between rings A and B, but substantial structural differences are present at positions 11β, 17α and 17β, as shown in Figure 1 and Figure 2. These significant structural differences indicate major variations in the pharmacodynamic, pharmacokinetic, and toxicological characteristics of these PR modulators.

## 3. Preclinical Studies

It is somewhat difficult to compare the preclinical characteristics of three different compound classes: a pure progestin without any PR-antagonistic activities (DNG), a pure antiprogestin (Vilaprisan), and a progestin with some anti-progestational characteristics (Asoprisnil and EC313) [33]. However, this approach is possible and can provide a clearer explanation of the peculiarities of each class in vivo and in clinical situations. The main results of the in vitro and in vivo preclinical studies are documented for DNG by Oettel et al. [34,35], for Vilaprisan by Wagenfeld et al. [36], for Asoprisnil by Elger et al. [23], DeManno et al. [37] and Chwalisz et al. [38], and for EC313 by Błaszczak-Świątkiewicz et al. [28]. Table 1 shows the relative receptor-binding affinities of different progestins and SPRMs. The 17α-cyanomethyl group of DNG is preferably tolerated by the progesterone receptor (PR) and contributes to the high selectivity of receptor binding. This is indicated by negligible binding affinities for the oestrogen receptor (ER), glucocorticoid receptor (GR), mineralocorticoid receptor (MR) and androgen receptor (AR) [39]. In contrast to the progestin DNG, the mesoprogestins Asoprisnil and EC313 demonstrate both agonistic and antagonistic PR activity, with a lack of or minor binding affinity to ER, GR, MR and AR [28,37].

## 4. In Vitro Findings

### 4.1. Dienogest (DNG)

In human uterine cytosol, the progesterone receptor (PR) exhibits a low binding affinity for DNG (10%), which is much lower than the affinity for the reference progesterone (100%). Furthermore, DNG only binds to the androgen receptor (AR). The affinity of DNG for oestrogen receptors (ERs), glucocorticoid receptors (GRs), and mineralocorticoid receptors (MRs) is negligible [39]. The discrepancy between moderate receptor binding and potent progestational activity in the endometrium is explained by the fact that a significant proportion of plasma DNG (approximately 10%, compared to 1–2% for other progestins) is available in the serum as free, biologically active DNG. This distinguishes DNG from other progestins [35]. The agonistic and/or antagonistic activity of DNG on human PR, AR, GR, MR, ERα and ERβ was measured using transactivation assays in several repeated studies. DNG activates PR (EC50 = 3.4 or 10.5 nmol/L). This progestin exhibits no agonistic activity on the AR but shows antagonistic activity (EC50 = 420.6 or 775.0 nmol/L). It exhibits no agonistic or antagonistic activity on GR and MR at concentrations up to 3000 nmol/L. DNG does not activate either ERα or ERβ (up to 3000.0 nmol/L). The estimated plasma concentration of DNG at ED50 (3.66 nmol/L) was close to the value required to activate PR. Thus, the stronger oral activity of DNG can be explained by its in vitro potency with regard to PR, its very high oral bioavailability (95%) and the high proportion (10%) of the biologically active, free, unbound compound in the serum [40]. Compared with the ratios observed in eutopic endometrium, a decreased PRB/PRA ratio and an increased ERβ/ERα ratio were demonstrated in ectopic endometriotic lesions derived from women with endometriosis. Treatment with DNG increased the PRB/PRA ratio and decreased the ERβ/ERα ratio. DNG also reduces YAP1 (yes-associated protein 1), which inhibits progesterone resistance in vitro and in vivo [41,42]. Therefore, DNG may improve progesterone resistance in endometriotic tissue in clinical practice by increasing the expression of PR-B and decreasing the expression of ERβ [41]. The non-genomic actions of DNG are of particular interest because the specifics of the pharmacodynamic profile depend on interactions via membrane PRs (mPRs). These interactions take place within seconds to minutes, as opposed to the genomic effects, which act over the course of hours [43,44]. Examples of the non-genomic effects of DNG are [4]:

- Inhibition of PGE2;
- Reduction in COX-2;
- Reduction in SDF-1 (stromal cell-derived factor 1; CXCL12);
- Inhibition of aromatase;
- Down-Regulation of VEGF;
- NF-ϏB inactivation.

Table 1 shows the relative receptor-binding affinities of different progestins and selected SPRMs. The 17α-cyanomethyl group of DNG is preferably tolerated by PR and contributes to the high selectivity of receptor binding. This is indicated by negligible binding affinities for the oestrogen receptor (ER), the glucocorticoid receptor (GR), the mineralocorticoid receptor (MR) and the androgen receptor (AR) [39]. In contrast to the progestin DNG, the mesoprogestins Asoprisnil and EC313 demonstrate both agonistic and antagonistic PR activity, with a lack of or minor binding affinity to ER, GR, MR and AR. Consequently, these mesoprogestins also demonstrate high PR selectivity [28,37].

### 4.2. Vilaprisan

Vilaprisan exhibited relative binding activity (RBA) to human PR of 41% ± 4.8%. Its affinity to GR was moderate to weak, and to AR it was low. Binding to ERα/β and MR could not be detected within the tested dose range [36]. To determine the agonistic and antagonistic activity of Vilaprisan, transactivation assays were carried out using cells that stably or transiently express PR (PRA or PRB, respectively). To create a positive control for reporter gene induction, the cells were exposed to promegestone (R5020). To inhibit reporter gene transcription, cells were treated with increasing concentrations of Mifepristone. Neither PRA nor PRB agonistic activity could be detected. Vilaprisan exhibited potent PR antagonist activity, with IC50 values of around 0.09 nmol/L and 100% efficacy for both PRA and PRB [36].

### 4.3. Asoprisnil

The competitive binding of Asoprisnil and its metabolite J912 to the following steroid receptors in cytosolic fractions of target tissues was tested: progesterone and estrogen (rabbit uterus), androgen (rat prostate), glucocorticoid (rat thymus) and mineralocorticoid (rat kidney). Compared to the natural ligand progesterone, Asoprisnil exhibited a binding affinity that was three times greater for PR. J912 also had a higher binding affinity than progesterone to PR. Both Asoprisnil and J912 demonstrated moderate binding affinities for the glucocorticoid receptor (GR) and low affinity for the androgen receptor (AR). No binding affinity to the estrogen receptor (ER) or mineralocorticoid receptor (MR) was detected for either compound [37,38]. The T47D breast cancer cell line was used in transactivation assays to determine PR-agonistic and PR-antagonistic activities. In this system, Asoprisnil demonstrated slightly less progesterone antagonistic activity than Mifepristone. The mean relative antiprogestational activity of Asoprisnil compared to Mifepristone was 69% ± 29% (*n* = 6). Asoprisnil showed no agonistic activity in this cell system. Anti-glucocorticoid activity was assayed in two cell lines (human ZR75 and rat H4-II-E), which contain glucocorticoid response elements. Asoprisnil demonstrated less than 10% of the anti-glucocorticoid activity of Mifepristone in these cell lines. The activity of aromatase (CYP19) in human placental microsomes was measured by determining the conversion of the substrate androstenedione to estrone. In this system, neither Asoprisnil nor J912 inhibited aromatase activity when tested at concentrations ranging from therapeutic plasma levels to well above those expected in human plasma (10–1000 ng/mL Asoprisnil and 20–2000 ng/mL J912). Similar to Uliprostal, Asoprisnil increases the PRA/PRB receptor ratio. This is achieved by decreasing PRB receptor levels and increasing PRA expression. This was observed in cultured leiomyoma cells. Significant changes in PR isoform content were also observed in normal myometrial cells [45,46]. The study of the endometrium after treatment with Asoprisnil revealed downregulation of stromal PR expression and upregulation of glandular PR expression. There was also a significant decrease in the number of uterine natural killer (NK) cells. These observations following the administration of an SPRM support the idea that the IL-15 pathway plays a role in the complex interplay between endometrial stromal cells, uterine NK cells and spiral arteries. It also appears to affect both physiology and heavy menstrual bleeding (HMB) [47]. In normal cycling endometrium, IL-15 levels respond to progesterone.

## 5. In Vivo-Findings

### 5.1. Secretory Transformation of Endometrium

The McPhail assay is the standard assay used to determine the progestational activities on the endometrium [48]. The extent of progestational activity can be measured using a histological score from 0 to 4 (a semiquantitative assay) in the secretory transformation of the 17β-estradiol-primed endometrium of immature female rabbits, independent of the administration route (oral, subcutaneous or local into the uterus lumen). To characterise the interactions of DNG with the rabbit endometrium, a total of 15 assays were carried out. The C19-norprogestin DNG exhibits strong PR-agonistic activity in comparison to the C21-progestin Chlormadinone acetate (CMA) and is much stronger than the well-known 19-norprogestins Levonorgestrel (LNG) and Norgestrel acetate (NETA). Following oral administration, the ED50 values (McPhail score 2) ranged from 0.02 to 0.05 mg/kg/b.w. over four days for both DNG and CMA, and from 0.11 to 0.45 mg/kg/b.w. for LNG. No dose–response relationships were found for NETA. This is due to the partial hepatic conversion of NETA to the potent oestrogen—ethinyl estradiol [49,50]. The values after subcutaneous administration were 0.02 mg/kg/b.w. for DNG and CMA, 0.05 mg/kg/b.w. for Levonorgestrel, 0.2 mg/kg/b.w. for NETA and 0.5 mg/kg/b.w. for progesterone over four days. Additionally, a single local intrauterine instillation of DNG did not improve the results. Simultaneous subcutaneous administration of progesterone did not demonstrate the antiprogestational action of orally or subcutaneously applied DNG. Unlike DNG, Vilaprisan exhibited complete antagonism of progesterone-induced differentiation of rabbit endometrial glands at a dose of 1 mg/kg/b.w. orally or higher. However, it did not exhibit agonistic activity at a dose of 10 mg/kg/b.w. [36]. Unlike DNG and Vilaprisan, Asoprisnil displayed dose-related progesterone receptor (PR) agonistic activity in the absence of progesterone (0.03–30 mg/kg b.w., s.c.), with a maximal McPhail score of 3.2 at 10 mg/kg/b.w., s.c. This response decreased with an extremely high dose of 100 mg/kg/b.w., s.c. (mean McPhail score 1.9), indicating a biphasic feature. These data suggest that Asoprisnil exhibits PR agonistic activity at low doses and that its activity threshold is lower than that of progesterone. This means that the maximum response with Asoprisnil is less than that with progesterone alone. When co-administered with progesterone (1.0 mg/kg/b.w., s.c.), Asoprisnil (0.03–30 mg/kg/b.w., s.c.) inhibited progesterone-induced changes in a dose-dependent manner. In rabbit uterine epithelium and in the uterus and vagina of guinea pigs, Asoprisnil and its metabolite exhibited only partial PR agonist activity. Asoprisnil did not completely block endometrial changes at any of the tested doses. In contrast, the pure antagonist Mifepristone (≥3 mg/kg//b.w., s.c.) effectively inhibited the progesterone-induced response [37]. The results from the McPhail assays underline that DNG has a profile similar to a pure PR agonist, Vilaprisan works like a pure antagonist, and Asoprisnil shows mixed agonistic/antagonistic activities (Table 2).

The dose–response curve of Asoprisnil and EC313 for both agonistic and antagonistic activities indicates that these mesoprogestins stabilise the function of PR at an intermediate activity level in the rabbit uterus [28,38].

### 5.2. Influence on Pregnancy

Progesterone is an essential hormone for maintaining pregnancy in mammals. Therefore, the anti-progestational activity of a given compound is used to determine the pregnancy-interruption model. In this regard, a PR antagonist competitively blocks the PR in the endometrium during pregnancy, ultimately leading to abortion at all stages. DNG, which was tested in rats (s.c.) and rabbits (p.o.), accelerates the passage of ova/blastocysts through the tubes but has no influence on implantation. When DNG is administered subcutaneously to rats after implantation (on day 5 after insemination), no influence on pregnancy is detected. In line with the findings in rats and rabbits, DNG administered orally on day 1 or days 1–3 of pregnancy (i.e., pre-implantation) also had no effect on pregnancy in guinea pigs [34,51]. Using the blastocyst transfer technique involving treated female rats and non-treated recipient rats, no direct embryotoxic effect of DNG was observed [52].

In the case of Vilaprisan, which was tested in rats on days 5–7 post coitum, pathological implantation sites were detected in two out of six animals at a dose of 0.15 mg/kg/b.w. administered orally. Full termination of pregnancy was observed at a dose of 1.5 mg/kg/b.w or higher, administered subcutaneously or orally [36].

DeManno et al. presented a study in which rats were administered the mesoprogestin Asoprisnil or Mifepristone subcutaneously or orally on days 5–7 of pregnancy. Asoprisnil and Mifepristone inhibited implantation in a dose-dependent manner. Oral doses of Asoprisnil and Mifepristone were equally effective (100% inhibition at 3 mg/kg/b.w.). In contrast to these rat findings, the results of a study involving guinea pigs in late pregnancy are presented in Table 3 below.

Whereas Mifepristone showed the same abortive potential in this model as in humans, it has been demonstrated that the mesoprogestin Asoprisnil and its main metabolite J912 are ineffective in inducing labour in this species. Note that, unlike mice, rats, hamsters, guinea pigs and humans have an interstitial type of implantation [53,54,55].

## 6. Preclinical In Vivo Model of SPRMs in Endometriosis

### 6.1. Dienogest (DNG)

Some in vivo models for endometriosis were developed. In the rat model, after s.c. transplantation of endometrial tissue and daily oral administration of 0.3 mg DNG/kg/b.w. or 3.0 mg Danazol/kg/b.w over 6 weeks, there were no significant differences in reducing the area of the autograft transplants and their secretory activity between these two tested groups in contrast to the control, although DNG was dosed ten times lower. Fischer et al. [56] investigated the effects of DNG on surgically induced endometriosis in rats after repeated oral administration. 0.3 mg/kg/b.w. significantly reduced the total endometrial lesion area, with an effect equivalent to much higher doses of Danazol 100 mg/kg/b.w. DNG at a dose of 0.3 mg/kg/b.w had no effect on uterine horn weight, indicating an absence of estrogenic effects. But, increasing the dosage to 1 mg/kg/b.w., the uterus weight was elevated (typical for 19-norprogestins). In the rat endometrial autograft model, DNG inhibited angiogenesis of the ectopic tissue, with confirmed structural changes in micro-vessels [57,58]. Finally, experimental endometriosis was induced in female rats at the proestrus stage via auto transplantation of endometrial tissue into the renal subcapsular space. Tested GnRH-antagonist Linzagolix (50 mg/kg/b.w.) and DNG (1 mg/kg/b.w.) significantly decreased cyst volume compared with the control group [59].

In rabbits, the endometrial transplants were placed on the mesometrium. The daily oral administration of 0.1; 1.0; or 10 mg/kg/b.w. indicated a biphasic preventive efficiency of DNG as well as Danazol for the secretory activity of autograft transplants [54]. In experimental endometriosis in rats, Katsuki et al. [55] found that DNG (0.1–1.0 mg/kg/BW orally) reduced the endometrial implant volume to the same extent as the much higher dose of Danazol (100 mg/kg/BW p.o.). DNG simultaneously ameliorated the endometrial implant-induced alterations of the immune system; it increased the natural killer activity of peritoneal fluid cells and splenic cells and decreased the number of peritoneal fluid cells and interleukin-1β production by peritoneal macrophages. In contrast, danazol (100 mg/kg/BW, p.o.) and the GnRH analogue Buserelin (30 µg/kg/BW, s.c.) had no such immunological effects. Additionally, the combined administration of DNG and Buserelin suppressed the bone mineral loss induced by Buserelin alone. In vitro studies with DNG revealed an anti-proliferative effect on rat endometrial cells due to the inhibition of protein kinase C activity, as well as a partial progestational effect. Based on these findings, DNG appears to be a potent agent with different mechanisms of action than Danazol and available GnRH agonists for the treatment of endometriosis [55].

Experimental endometriosis in mice was induced by either intraperitoneal or subcutaneous endometrium transplantation using donors and recipients (three pieces of endometrial tissue were sutured to the vessels of the mesentery). For orientation, the suggested daily oral dose for females was: 5 mg for Ulipristal acetate, 20–30 mg for Dydrogesterone, and 2 mg for DNG. The dosage of each medication for the mice was calculated using an FDA-based formula that takes into account the surface area of humans and experimental animals [58]. It was found that Ulipristal acetate, Dydrogesterone, and DNG decreased lesion and transplant size as well as the expression of the proliferation marker PCNA (Proliferating-Cell-Nuclear-Antigen). The PR expression was significantly lower in all groups. Endometrial gland count in the uterus was significantly increased only in the DNG group. Upon treatment cessation, in the DNG group, the lesion quickly rebounded.

### 6.2. Vilaprisan

There are no reports about the influence of Vilaprisan on a preclinical endometriosis model. So far, its pharmacological activity has been extensively tested in a mouse xenograft model of human uterine fibroids [36]. Vilaprisan significantly reduced graft weight gain in a dose-dependent manner, by 65% and 95% compared to the control group at doses of 1.0 and 3.0 mg/kg/b.w., respectively.

It is important to distinguish between the direct effects of PR antagonists (such as Vilaprisan) and other SPRMs on the endometrium and endometriotic lesions and their overall systemic effects. The PR-antagonists exert a direct anti-proliferative effect on the endometrium, referred to as non-competitive anti-oestrogenic action. With regard to systemic effects, reducing estradiol secretion to normal levels in the follicular phase of the menstrual cycle can prevent unwanted side effects such as symptoms of estrogen deficiency [9,33,60].

### 6.3. Asoprisnil

The unique and selective effects of SPRMs on the endometrium are specific to menstruating primates such as Old World monkeys. Therefore, cynomolgus and rhesus macaques are suitable models for testing SPRMs, as the hormonal regulation and morphological changes in their endometrium during the menstrual cycle are similar to those of humans [61]. Asoprisnil exhibits direct, tissue-specific anti-proliferative effects that target only the endometrium. These findings were not anticipated. The effect of Asoprisnil on endometrial morphology was studied in cynomolgus monkeys that received oral doses of 10, 30 or 90 mg/kg/b.w for 90 days. To enhance oral absorption, Asoprisnil was suspended in a vehicle containing 10% ethanol, 35% polyethylene glycol (PEG 300) and 55% poloxamer 157 (Cremophor EL). All doses suppressed the proliferation markers Ki-67 and phospho-H3 in the endometrial glands. The two higher doses caused significant shrinkage in endometrial thickness without inducing progestational effects such as glandular sacculation and secretion. There was a trend towards stromal compaction, but no evidence of spiral artery degeneration. Studies on animals, including non-human primates, have shown that Asoprisnil blocks ovulation, induces amenorrhoea and suppresses endometrial proliferation in the presence of normal serum oestrogen concentrations. The mesoprogestin Asoprisnil did not suppress estrogen action in other parts of the reproductive tract, such as the oviduct and vagina. These preclinical studies demonstrate Asoprisnil’s potential to control prostaglandin production in the endometrium, which is believed to contribute to endometriosis-related pain in women, and to alleviate endometriosis symptoms. The preclinical findings in non-human primates are very similar to the results in humans [24].

## 7. Clinical Studies

Only a few studies have been published on the treatment of endometriosis with Mifepristone [62,63,64] and Uliprostal acetate [65]. However, these regimens have not been implemented in practice and are therefore not relevant to this discussion.

### 7.1. Dienogest (DNG)

Dienogest (DNG) is a progestin that has been characterised in detail in terms of molecular biology, biochemistry, pharmacokinetics, endocrine and safety pharmacology as well as toxicology [66]. The progestin is specifically approved for the therapy of endometriosis. Therefore, DNG can be the standard for the comparison of the therapeutic potential of other forms of PR-modulation. DNG possesses high endometrial and anti-endometriotic effects and moderate inhibition of gonadotrophin secretion demonstrated in many clinical studies. DNG (STS557) was also included in the Human Reproduction Program of the World Health Organization (WHO) [66,67].

#### 7.1.1. Pivotal Studies Phase II (Dose Finding)

##### In Europe

Köhler et al. [68] conducted an open-label, dose-ranging study to evaluate the efficacy and safety of 1, 2 and 4 mg of DNG administered daily to women with histologically confirmed endometriosis (*n* = 68). Efficacy was assessed using second-look laparoscopy and patient-reported symptoms. DNG reduced mean revised American Fertility Society (AFS) scores from 11.4 to 3.6 (*n* = 29; *p* < 0.001) in the 2 mg group and from 9.7 to 3.9 (*n* = 35; *p* < 0.001) in the 4 mg group. Both DNG doses were generally well tolerated, with low rates of treatment discontinuation due to adverse events. The 1 mg arm was discontinued due to inadequate bleeding control. The authors concluded that 2 mg of DNG once daily is the optimal dose.

Klipping et al. [69] investigated the ovulation-inhibiting effects of DNG (Visanne^®^) in a randomised, dose-controlled, pharmacodynamic trial involving healthy women. DNG was administered at doses of 0.5, 1, 2 or 3 mg daily for up to 72 days to women aged 18 to 35 years (*n* = 102). Ovarian activity was assessed during the pre-treatment period and two treatment periods (days 0–36 and days 37–72) using the Hoogland-Skouby score, which is based on follicular size and serum estradiol and progesterone levels. Hoogland-Skouby scoring indicated ovulation in all women during the pre-treatment period, decreasing to three out of 21, one out of 23, zero out of 20 and zero out of 23 women in the 0.5-, 1-, 2- and 3 mg groups, respectively. Maximum serum estradiol concentrations were similar to pre-treatment levels in the 0.5 mg and 1 mg groups, and decreased moderately (within physiological levels) in the 2 mg and 3 mg groups. Endometrial thickness was reduced by all DNG doses. Hormonal changes observed during follow-up indicated resumption of ovulation in most women shortly after treatment cessation. Daily DNG doses of ≥2 mg provide moderate suppression of 17β-estradiol production and reliable ovulation inhibition, which reverses rapidly after treatment cessation.

##### In Asia, in Japan

The peculiarity of Japanese studies is that 1 mg DNG is given orally twice daily. But the total daily dose of 2 mg is always given. Harada and Taniguchi [70] published the results of a randomised, double-blind, multicentre, parallel-group study designed to confirm the dose–response relationship in the efficacy and safety of DNG in patients with endometriosis. A total of 187 women diagnosed with endometriosis were randomised into three groups and administered oral DNG at doses of 1, 2 or 4 mg for 24 weeks. The primary efficacy endpoint was global efficacy, an overall assessment of the improvement in seven endometriosis symptoms and signs (lower abdominal pain, lumbar pain, defecation pain, dyspareunia, pain during internal examination, induration in the pouch of Douglas, and limited uterine mobility) at the end of treatment. The proportion of patients who improved in terms of global efficacy was 63.8%, 66.7% or 73.2% for total treatment of 1, 2 or 4 mg, respectively. No statistically significant dose–response relationship was found. The proportion of patients assessed as tolerable in terms of global safety was 85.2%, 95.0% and 82.3% for the 1 mg, 2 mg and 4 mg treatment groups, respectively. Furthermore, no significant difference was found. The most common76 adverse event was uterine bleeding, which was observed with a similar frequency and severity in all three groups. However, the bleeding was well tolerated in all groups. Serum estradiol levels did not change in the 1 mg group but decreased significantly in the 2 and 4 mg groups. Additionally, the mean estradiol level in the 2 mg group (37.4 pg/mL) was within the recommended range (30–50 pg/mL) of the ‘therapeutic window’ theory for the efficacy and safety of medical treatment for endometriosis. However, it was below this range in the 4 mg group (26.2 pg/mL). The authors concluded that a total dose of 2 mg of DNG would be suitable for treating endometriosis, as the global efficacy, tolerability, and safety of DNG were confirmed.

#### 7.1.2. Pivotal Studies Phase III

##### In Europe

Strowitzki et al. [71] published the results of a 12-week, randomised, double-blind, placebo-controlled, multicentre (33 centres) study conducted in Germany, Italy and Ukraine. The study included 198 women aged 18–45 years with laparoscopically confirmed endometriosis and an endometriosis-associated pelvic pain (EAPP) score of at least 30 mm on a visual analogue scale (VAS). DNG 2 mg or a placebo was administered orally once daily. The primary efficacy variable was the absolute change in EAPP score from baseline to week 12, as determined by the target variables of change in VAS score and change in supportive analgesic medication (ibuprofen) intake for pelvic pain. The mean reduction in VAS score between baseline and week 12 in the full analysis set was 27.4 mm and 15.1 mm in the DNG and placebo groups, respectively—a significant score difference of 12.3 mm in favour of DNG (*p* < 0.0001). Changes in supportive analgesic medication intake were modest in both groups. The primary efficacy measure of absolute change in EAPP demonstrated the superiority of DNG over placebo. DNG was generally well tolerated, with few adverse events associated with the therapy. Strowitzki et al. [72] found that DNG is as effective as the GnRH analogue Leuprolide acetate (LA) in treating painful endometriosis symptoms in a 24-week randomised multicentre open-label trial. Patients with confirmed endometriosis were randomised to receive treatment with either DNG (2 mg/day orally; *n* = 124) or LA (3.75 mg depot intramuscular injection every 4 weeks; *n* = 128) for 24 weeks. The primary efficacy variable was the absolute change in pelvic pain from baseline to the end of treatment, as assessed using a visual analogue scale (VAS). 87.9% and 93.8% of the respective groups completed the trial. Absolute reductions in VAS score from baseline to week 24 were 47.5 mm with DNG and 46.0 mm with LA, demonstrating the equivalence of DNG to LA. Hypoestrogenic effects (e.g., hot flushes) were reported less frequently in the DNG group. As expected, bleeding episodes were suppressed less with DNG than with LA. Changes in mean lumbar BMD between screening and final visit were +0.25% with DNG and −4.04% with LA (*p* = 0.0003). Markers of bone resorption increased with LA but not with DNG. Therefore, DNG offered advantages in safety and tolerability.

Petraglia et al. [72] reported the open-label extension study which followed the above-mentioned study by Strowitzki et al. [73] where 168 women were enrolled. The study was extended for up to 53 weeks. All women received DNG 2 mg/day orally. The follow-up completion rate among women was 90.5% (*n* = 152). A significant decrease in pelvic pain was observed during continued DNG treatment (*p* < 0.001). The mean frequency and intensity of bleeding decreased progressively. Adverse events, which were generally rated as mild or moderate, led to the withdrawal of only four patients (2.4%). No clinically relevant changes in laboratory parameters were observed. During the treatment-free follow-up period (*n* = 34), the reduction in pelvic pain persisted while the frequency and intensity of bleeding returned to normal levels.

All three European Phase III pivotal studies plus the pivotal Phase II dose-finding [69] were pooled and analysed for safety and tolerability by Strowitzki et al. [69,72]. This pooled analysis of 332 women with endometriosis showed that dienogest was well tolerated, demonstrating a favourable safety profile over a period of up to 65 weeks. The most common adverse drug reactions were headache, breast discomfort, depressed mood and acne, each of which occurred in fewer than 10% of women. These adverse events were generally mild to moderate in intensity and associated with low discontinuation rates. Additionally, oestrogen (E2) levels remained within the low physiological range, consistent with previous findings suggesting that Dienogest 2 mg exhibits therapeutic efficacy without causing estradiol deficiency [73].

##### In Asia, in Japan

Harada et al. [74] conducted a randomised, open-label, multicentre, controlled trial to compare the efficacy and safety of oral DNG and the intranasal GnRH agonist Buserelin acetate (BA) in patients with endometriosis. This Phase III study was conducted in 24 centres in Japan. A total of 271 patients with endometriosis were included in the study and treated with either DNG (one 1 mg tablet taken morning and evening with meals every day) or BA (900 µg/day, administered intranasally) for 24 weeks. The main outcome measures were the pre- to post-treatment changes in the scores of five subjective symptoms during non-menstruation (lower abdominal pain, lumbago, defecation pain, dyspareunia, and pain on internal examination) and two objective findings (induration in the pouch of Douglas and limited uterine mobility). DNG reduced the scores of all symptoms and findings by the end of the treatment period. The mean changes in the scores of all symptoms and findings, except for induration in the pouch of Douglas, were comparable with those obtained with BA. The two groups showed similar changes in VAS scores for lower abdominal pain and lumbago from baseline to the end of treatment. The mean reduction (± standard deviation) from baseline to the end of treatment in the DNG group was −30.2 (±31.8) for lower abdominal pain and −15.7 (±28.7) for lumbago, whereas the corresponding values for the BA group were −27.3 (±33.8) and −17.3 (±24.8). Compared with BA, DNG was associated with more frequent irregular vaginal bleeding (DNG: 95% vs. BA: 67%) and fewer hot flushes. The reduction in bone mineral density (BMD) during DNG treatment was significantly lower than during BA treatment. The authors concluded that DNG is as effective as intranasal BA in alleviating endometriosis and results in less BMD loss (−1.0 ± 2.3% vs. −2.6 ± 2.3%; percent change from baseline to the end of treatment). The mean serum concentrations of estradiol at baseline and at week 16 of treatment were 86 ± 60 pg/mL and 38 ± 56 pg/mL, respectively, in the DNG group. The corresponding values in the BA group were 87 ± 61 pg/mL and 21 ± 38 pg/mL. The mean serum concentrations of CA125 at baseline and at the end of treatment were 65.5 U/mL and 41.5 U/mL, respectively, in the DNG group, whereas the corresponding values were 58.3 U/mL and 28.6 U/mL in the BA group. Momoeda et al. [75] investigated the safety and efficacy of 52 weeks of treatment with DNG in patients with endometriosis. A total of 135 patients received 1 mg of DNG twice daily, starting on days two to five of their menstrual cycle. Adverse drug reactions and bone density were evaluated. Global improvement was assessed based on changes in the severity categories of five subjective symptoms (lower abdominal pain, lumbago, dyschesia, dyspareunia and pain during a vaginal examination) and two objective findings (induration of the pouch of Douglas and limited uterine mobility) during the non-menstrual period. The most common adverse drug reactions were metrorrhagia (71.9%), headaches (18.5%), and constipation (10.4%). No clinically significant changes were noted in the incidence or severity of reactions associated with long-term DNG use (52 weeks). Changes in bone mineral density (BMD) of the lumbar spine, as measured by dual-energy X-ray absorptiometry, were −1.6% ± 2.4% and −1.7% ± 2.2% (mean ± standard deviation) at 24 and 52 weeks respectively. These were statistically significant decreases; however, there was no cumulative decrease. The proportion of patients assessed as having experienced marked or moderate improvement in terms of global improvement was 72.5% (95/131 cases) at 24 weeks and 90.6% (106/117 cases) at 52 weeks. Conclusion: The long-term effect of DNG on BMD was limited, whereas efficacy increased cumulatively.

##### In China

The first placebo-controlled, randomised, double-blind Phase III study came from China [76]. This multicentre, 24-week study (*n* = 23) evaluated the efficacy and safety of 2 mg of DNG administered once daily to 255 Chinese women aged 18–45 years with laparoscopically diagnosed endometriosis and an EAPP score of at least 30 mm on a 0–100 mm visual analogue scale. The primary efficacy variable was the absolute change in EAPP score from baseline to week 24. Secondary efficacy variables included proportions of responders and intake of supportive analgesic medication. Safety variables included adverse events (AEs), laboratory parameters and bleeding patterns. Bone mineral density (BMD) was evaluated in a subset of 140 women. After 24 weeks of treatment, the mean reduction in EAPP differed significantly between the treatment groups (DNG vs. placebo; *p* < 0.0001). Secondary efficacy analyses supported the significant superiority of DNG over placebo. DNG was well tolerated, with few AEs associated with the therapy. DNG had no effect on BMD levels after 24 weeks of treatment. This study was the basis for the approval of Visanne in China in 2018. The follow-up was published by Yu et al. [77].

Finally, a large, prospective Korean study should be cited [78]. This study included 3356 patients with endometriosis from 73 centres. All patients were treated with 2 mg of DNG and monitored for at least six months following their initial visit. The effectiveness of DNG in alleviating pain was assessed by comparing visual analogue scale (VAS) scores at the end of the follow-up period with baseline scores. The most commonly reported adverse events were abnormal uterine bleeding (4.14%, *n* = 129), weight gain (2.57%, *n* = 80), and headaches (1.22%, *n* = 38). The proportion of patients with favourable bleeding patterns increased with treatment duration. Amenorrhoea was observed in 29.63% of patients at the three-month follow-up, 41.25% at the six-month follow-up, 46.26% at the 12-month follow-up, and 53.20% at the follow-up after 12 months. The mean (±SD) VAS change from baseline at the final follow-up visit was −28.19 ± 28.39 mm (*p* < 0.0001). The authors concluded that this large cohort study confirms that DNG is safe and effective for the treatment of endometriosis in routine clinical practice.

## 8. Summary of Clinical Evidence

### 8.1. DNG

Dienogest (DNG), a non-ethinylated 19-nortestosterone derivative, has strong endometrial activity. This progestin shows low binding to the androgen receptor and almost negligible binding to the oestrogen receptor, glucocorticoid receptor, mineralocorticoid receptor and also to SHBG. An oral dose of 1 mg/day is required to inhibit ovulation in cycling women. DNG inhibits the growth and cytokine production of endometriotic cells. Maximum serum DNG concentrations are reached within approximately two hours, and the specific profile of DNG is based on both genomic and non-genomic actions [4]. Based on dose-efficacy relationships, 2 mg/day of DNG is preferred [79].

In all studies, the revised American Fertility Society (r-AFS) scores or visual analogue scale (VAS) indicated a significantly high efficacy in the same range as that of GnRH analogues (Leuprolide acetate, Triptorelin, Buserelin and goserelin); see Section 7.1.2.

In comparison to the standard pharmacological treatment regimen for endometriosis (GnRH analogues, danazol and other progestins), the tolerability, including laboratory findings, was excellent (e.g., bone mineral density (BMD), hot flushes), and it was suitable for long-term use in many patients with appropriate clinical monitoring. Overall, these studies demonstrated that DNG is not inferior to GnRH analogues but has significantly fewer side effects, such as oestrogen deficiency. An additional dose of estradiol and norethisterone acetate (NETA) seems to be necessary for oral GnRH analogues [80]. Seo et al. presented unexpected findings [81]. In a retrospective cohort study, DNG increased the reoperation rate, but only from the tenth treatment month onwards. This result contrasts with that of Ceccaroni et al. [82], who found in a prospective randomised controlled trial that DNG is as effective as GnRH analogues in preventing the recurrence of deep infiltrating endometriosis. In a retrospective study, Koshiba et al. [83] also found that early DNG therapy after recurrence of postsurgical ovarian endometrioma appears to reduce the risk of repeated surgery.

Overall, DNG is an effective long-term treatment option for endometriosis (SOGC Clinical Practice Guideline) [84,85]. A high-dose pilot study (DNG 20 mg/day) demonstrated that, after 24 weeks of treatment, the total R-AFS score had decreased by 59% from baseline. Even at this high dosage, tolerability was excellent. Another advantage of DNG is that it increases the progesterone receptor isoform B/A ratio in patients with ovarian endometriosis. This is important for overcoming progesterone resistance. DNG treatment increases the PR-B/PR-A ratio and decreases the ERβ/ERα ratio in patients with endometriomas [41]. In addition to treating endometriosis, DNG is also clinically effective for treating adenomyosis and uterine myomas/fibroids [15,16].

Meanwhile, some generic versions of 2 mg DNG (Visanne^®^), such as Endovelle^®^, developed in Europe, are on the market, as well as separate developments in China [86]. The Japanese company Mochida sells Dinagest^®^ (1 mg of DNG twice daily).

### 8.2. Vilaprisan

The development of Vilaprisan was focused primarily on the treatment of uterine fibroids and the associated heavy uterine bleeding. In this sense, 9 successful clinical studies are discussed in the literature including the influence of Vilaprisan on ovarian function [5,87,88,89,90]. Phase 2 studies showed that administering 2 mg of Vilaprisan daily led to effective control of heavy menstrual bleeding, marked reductions in fibroid volume and improvements in patients’ health-related quality of life, with favourable tolerability. Thus, this dose was selected for Phase 3 studies [33]. No differences were seen in pharmacokinetics between Caucasian and Chinese women [91]. Moeller et al. and Schultze-Mosgau et al. [34,92] provided characteristics of Vilaprisan’s safety and efficacy profile, showing strong exposure–response relationships in patients with uterine fibroids experiencing heavy menstrual bleeding. Vilaprisan was well tolerated at all tested doses. Based on its favourable pharmacokinetic and pharmacodynamic profile, the same 2 mg/day dose can be used for all patients, irrespective of body weight, race or age. It can also be used for patients with mild or moderate renal or hepatic impairment [90]. Vilaprisan entered Phase III clinical trials for endometriosis treatment. However, all clinical studies of Vilaprisan were suspended due to the discovery of drug toxicity signals in long-term animal tests conducted in parallel with Phase III clinical trials, despite subsequent Phase II clinical data demonstrating the drug’s safety and tolerability [13]. Based on the review of presented papers, endometriosis-specific clinical evidence of Vilaprisan is limited.

### 8.3. Asoprisnil

Chwalisz et al. [25,26] conducted a multicentre, double-blind, placebo-controlled, parallel-group, phase II study to evaluate the safety and efficacy of three doses of Asoprisnil (5 mg, 10 mg, and 25 mg) administered over a period of 12 weeks in women with laparoscopically confirmed endometriosis and moderate-to-severe baseline pain. All three doses significantly reduced average daily combined non-menstrual pelvic pain and dysmenorrhoea scores compared to placebo at all treatment months (*p* < 0.05). By month 3, mean pain scores decreased by approximately 0.5 in each of the three dose groups, compared to a decrease of less than 0.1 with placebo. Similar results were obtained for non-menstrual pelvic pain and dysmenorrhoea when analysed separately using daily diaries or monthly assessments during visits. This treatment also induced amenorrhoea in a dose-dependent manner throughout the entire treatment period (placebo: 0%; 5 mg: 50%; 10 mg: 71%; 25 mg: 93%). Meanwhile, Asoprisnil had no significant effect on E2 serum levels compared to those in the follicular phase of the menstrual cycle. Adverse events were evenly distributed among the treatment and placebo groups and were generally mild and self-limiting. No serious drug-related adverse events were reported during the treatment or follow-up period. The authors concluded that Asoprisnil was effective in alleviating the primary symptoms of endometriosis-related pain and that it was well tolerated during the three-month treatment period. As shown above, there were no laboratory or clinical signs of oestrogen deprivation. In normal cycling endometrium, IL-15 levels are progesterone-responsive. In the study of Asoprisnil-treated endometrium, there was downregulation of stromal PR expression and upregulation of glandular PR expression, as well as a marked reduction in the number of uterine NK cells. These observations following the administration of an SPRM support a role for the IL-15 pathway in the complex interplay between endometrial stromal cells, uterine NK cells and spiral arteries, as well as having an effect on both physiology and HMB [5]. Therefore, Asoprisnil may offer a novel, tissue-selective method of controlling endometriosis-related pain [26]. However, long-term treatment with Asoprisnil can trigger PRM-associated endometrial changes, and development was discontinued due to concerns regarding PAEC. Subsequent interpretation of these changes has evolved, though. These benign and reversible endometrial changes, which are also seen with other SPRMs, have been misinterpreted, resulting in the termination of the entire development programme [27].

## 9. Discussion

Endometriosis is a heterogeneous condition with multiple phenotypes and potentially different dominant mechanisms. It is one of the most common benign gynaecological disorders affecting women of reproductive age. Despite its enormous clinical and financial impact, pharmacological treatment remains unsatisfactory. This is because the aetiology and pathogenesis are largely unknown, at least in important parts. The lack of a uniform pharmacological target makes the search for effective compounds more difficult [4,10,18]. One radical scenario is that several different diseases with different aetiopathological causes are grouped under the term ‘endometriosis’. It is therefore not surprising that very different approaches are being tested and used clinically. The range of medications includes non-steroidal anti-inflammatory drugs (NSAIDs) and progestins as first-line drugs and combined oral contraceptives (OCs) and oral gonadotropin-releasing hormone antagonists (GnRH) as second-line drugs [13]. DNG is the only progestin specifically designed for treating endometriosis. This paper briefly discusses the preclinical and clinical features of the drug, excluding DNG toxicology and pharmacokinetics, as both fields are free of significant issues. DNG effectively alleviates pain symptoms associated with endometriosis, including dysmenorrhoea, premenstrual pelvic pain, painful intercourse, and chronic pelvic pain. It is primarily prescribed to patients with mild symptoms, but its therapeutic effect is limited in women with severe symptoms. Nevertheless, DNG is commonly used for long-term symptom management [13]. DNG has performed well in the steroid market. The prescription of this progestin has increased rapidly [89].

Taken together, DNG is the standard, so a new therapeutic principle of choice must demonstrate at least the same pharmacodynamic profile as this progestin. The following are the advantages of DNG:

- Reliable reduction in pelvic pain;
- Relative target specificity on endometrium incl. endometriotic lesions;
- Pronounced lesion-shrinking effects in a few cases;
- Favourable PRA/PRB quotient for reducing the progesterone-resistance;
- Advantageous non-genomic actions;
- Very good tolerability even in long-term treatment over years (SOGC Clinical Practice Guideline, 2010) [84].

The potential disadvantages of the DNG therapy are as follows:

- Reduced efficiency in severe cases of endometriosis;
- Uterine bleeding irregularities which can be improved by long-term administration;
- Limited E2 levels (lower physiological range);
- Gynaecologists should be aware of the potential impact of DNG on mood (like in other mono-therapies with progestins) [90].

Dienogest should be the standard against which pharmacological approaches involving steroids for the treatment of endometriosis are developed [14].

Shi et al. [13] described new approaches to the pharmacological therapy of endometriosis, such as orally bioavailable gonadotropin-releasing hormone antagonists (e.g., Elagolix, Linzagolix and SKI-2496), the selective oestrogen receptor modulator Bazedoxifene, prostaglandin E2 (PGE2) receptor subtype EP4 antagonists (e.g., BAY 1316957), aromatase inhibitors (e.g., Letrozole), dopamine receptor agonists (e.g., Quinagolide) and monoclonal antibodies targeting interleukin-33 (e.g., MT2990). It is difficult to assess the therapeutic value of these newcomers in the future. From the present point of view, the greatest chances can be attributed to three drug classes: GnRH antagonists, SERMs and aromatase inhibitors. The most important effect of these three categories is their anti-estrogenic mode of action. However, it is difficult to imagine that these drugs act specifically on endometriomas. The anti-estrogenic effect will be systemic, resulting in all the problems associated with a hypoestrogenic state.

In the past, the question arose as to whether the PR antagonist Vilaprisan and/or the PR mixed agonist/antagonist Asoprisnil could eliminate the shortcomings of DNG more effectively. Unfortunately, insufficient clinical studies have been conducted due to the early termination of the clinical development of both compounds to determine whether these compounds overcome the limitations of Dienogest.

## 10. Conclusions

The next step could be to increase the partial progestin-agonist effect of mesoprogestins. A comparison of Vilaprisan and Asoprisnil suggests that mesoprogestins exhibit greater tissue selectivity, targeting only the endometrium and inducing complete amenorrhoea. This is also an advantage over DNG. Therefore, further research into the mesoprogestin principle seems promising. Would the therapeutic efficacy of a combination of a PR agonist and a PR antagonist be enhanced by increasing the proportion of PR agonist activity?

Details of the extensive in vitro programme, including competitive binding and transactivation assays (PRA and PRB, GR, MR, AR, ERα and ERβ), induction of PR nuclear translocation and PRB-nuclear corepressor (NCOR) and tyrosine kinase Src interactions, can be seen in Blaszczak-Świątkiewicz et al. [28,70,93]. Furthermore, EC313 activates more PRB than PRA. This is consistent with findings relating to DNG but inconsistent with results relating to UA [45,46]. This could be helpful in treating progesterone resistance in human endometriotic tissue [41,42].

As with Asoprisnil, the expression of NF-κB and cyclin D1 is inhibited. Therefore, to optimise the mesoprogestin strategy, more attention should be paid to the interface between nuclear and membrane steroid signalling [70,84,94]. In the McPhail assay in rabbits, the partial significant PR agonistic effect on the endometrium was greater than the significant PR antagonistic activity (see Table 2). In guinea pigs, EC313 exhibits progesterone receptor dominance in the genital tract and inhibits the effects of unopposed oestrogen. Even very high doses (30.0 mg EC313/kg/b.w., s.c.) administered twice on pregnancy days 43 and 44 did not induce premature labour in the same species (unlike UPA, dosed at 10.0 and 30.0 mg/kg/b.w., s.c.). The anti-ovulatory activity of EC313 in guinea pigs exceeds that of Uliprostal acetate or Mifepristone. Following subcutaneous administration in rats, no oestrogenic, anti-estrogenic, androgenic, anti-androgenic, glucocorticoid or anti-glucocorticoid actions were observed. High target specificity was demonstrated in an endometriosis model involving human endometriotic transplants in immunodeficient mice. Significant anti-endometriotic effects were seen starting with a low dosage of 0.01 mg/kg/b.w. The same results were demonstrated in another xenograft model for treating humanised uterine fibroid tissue in immunodeficient mice. In these experiments, the lowest dose of 0.01 mg/kg/b.w. was also significantly active [28,93,95].

## Figures and Tables

**Table 1:** The relative receptor-binding affinity of different SPRMs.

	PR	ERs	GR	MR	AR
Progesterone	+	-	+	+	-
Gestodene	+	-	+	+	+
3-Keto-Desogestrel	+	-	+	-	+
Levonorgestrel	+	-	+	+	+
Drospirenone	+	-	-	++	-
Dienogest	+	-	-	-	-
Vilaprisan	+	-	~	-	-
Asoprisnil	+	-	~	-	-
EC313	+	-	~	-	-

- no or scarce effect; ~ moderate effect; + distinct effect; ++ strong effect.

**Table 2:** Comparison of McPhail results with progesterone and other SPRMs (daily dosages).

Compound	Score 2 for PR-Antagonistic Activity (mg/Animal/Day s.c.)	Score 2 for PR-Agonistic Activity (mg/Animal/Day s.c.)	Quotient Between Antagonistic and AGONISTIC Action
Progesterone	n/a, pure agonist	0.1	n/a
Dienogest	n/a, pure agonist	0.04	n/a
Vilaprisan	<0.3	n/a, pure antagonist	n/a
Asoprisnil	0.3	0.2	1.5
EC313	10.0	1.0	10.0

n/a—not applicable.

**Table 3:** Labour- inducing activity of the SPRMs: Asoprisnil and its metabolite J912; the antiprogestins Mifepristone and Onapristone in late pregnant guinea pigs [37].

Compound	ED_50_ (mg/Animal/Day)
Mifepristone	3.8
Onapristone	3.0
Asoprisnil	>100
J912	>100

## Data Availability

No new data were created or analysed in this study. Data sharing is not applicable to this article.
